# Effectiveness of Two Wavelengths of Diode Laser and Amorphous Calcium Phosphate–Casein Phosphopeptide Mousse in the Treatment of Dentinal Hypersensitivity: A Randomized Clinical Study

**DOI:** 10.1155/ijod/1257136

**Published:** 2024-11-22

**Authors:** Manjusha Nambiar, Bhavya Shetty, Ibrahim Fazal, Safiya Fatima Khan, Mehul A. Shah, Vignesh Kamath, Shahaziya Faruk, Vaishnavi Jalaj, Sowmya N.

**Affiliations:** ^1^Department of Periodontology, Sri Rajiv Gandhi College of Dental Science and Hospital, Bangalore, Karnataka, India; ^2^Department of Periodontology, Faculty of Dental Sciences, Ramaiah University of Applied Sciences, Bangalore, Karnataka, India; ^3^Department of Periodontics and Implantology, Brny Medical Complex, Al-Rashidiyah, Al-Ahsa, Saudi Arabia; ^4^Department of Periodontics, Al Wafa Dental Center, Unayzah, Qassim, Saudi Arabia; ^5^Department of Public Health Dentistry, Bharati Vidyapeeth (Deemed to be University) Dental College and Hospital, Pune, Maharashtra, India; ^6^Department of Prosthodontics and Crown and Bridge, Manipal College of Dental Sciences Mangalore, Manipal Academy of Higher Education, Manipal 576104, Karnataka, India; ^7^Manipal College of Dental Sciences Mangalore, Manipal Academy of Higher Education, Manipal 576104, Karnataka, India; ^8^Department of Prosthodontics and Crown and Bridge, Manipal College of Dental Sciences Mangalore, Manipal Academy of Higher Education, Manipal 576104, Karnataka, India

**Keywords:** diode laser, GC tooth mousse, numeric rating scale, tooth sensitivity

## Abstract

**Objective:** In office methods for immediate relief of dentinal hypersensitivity (DH) has long been an area of research. This study compared the efficacy of 660 nm diode laser, 980 nm diode laser, and amorphous calcium phosphate–casein phosphopeptide (ACP–CPP) agent in the treatment of DH.

**Materials and Methods:** A total of 39 patients with minimum three hypersensitive teeth in at least one quadrant were selected and randomly divided into three groups; Group A, B, and C patients were treated by 660 nm diode laser, 980 nm diode laser, and ACP–CPP agent, respectively. All the hypersensitive teeth were stimulated with tactile; thermal and air stimuli, and the pretreatment and posttreatment hypersensitivity scores were evaluated with the numeric rating scale (NRS) at baseline, 30 min, 1 week, 1 month, 3 months, and 6 months. The Shapiro–Wilk test was used to determine the uniformity of the data. The Chi-square (*χ*^2^) test of significance was used to compare proportions. For intergroup comparison, the Kruskal–Wallis test and the Mann–Whitney *U* test were utilized.

**Results:** At 30 min and 1 week, the 980 nm laser was more effective than the 660 nm laser, but there was no statistically significant difference between the two groups at 1, 3, and 6 months. Throughout the trial period, the 660 nm and 980 nm diode lasers were more effective than the ACP–CPP agent in lowering DH.

**Conclusions:** 660 nm diode laser, 980 nm diode laser, and ACP–CPP agent showed positive prospective as effective desensitizers when utilized as a clinical procedure.

## 1. Introduction

Dentinal hypersensitivity (DH) ranks among the most unpleasant and persistent periodontal disorders, featuring a projected frequency in the general population ranging from 4% to 57% and a greater prevalence during the third and fourth decades of life [1–8]. Though tooth sensitivity continues to be a main cause of concern amongst the dental fraternity; currently, there is no established standard of treatment for clinical management of DH. Various in-office methods for relief of hypersensitivity has long been an area of research. Owing to the immense advances in the research and development in the area of laser dentistry, lasers are now considered to be an effective tool in the dental armamentarium, while managing DH. Various therapeutic options of diode laser, ranging from low-level laser to high-power laser can be used in the treatment of dentinal pain. Also, demineralizing agents that has been derived from milk casein products such as amorphous calcium phosphate (ACP) and casein phosphopeptide (CPP) has been used in alleviating hypersensitivity pain [9–11]. However, no studies have been performed comparing the in-office therapeutic approaches such as diode laser and ACP–CPP agent in the management of DH. Therefore, we aimed to assess and compare the effectiveness of in-office treatment modalities like 660 nm diode laser, 980 nm Diode laser, and ACP–CPP agent in the treatment of hypersensitivity.

## 2. Materials and Methods

The study was designed as 6 months, single-center, single-blinded, and randomized clinical study. Thirty-nine individuals (25 men and 14 females) with at least three hypersensitive teeth in at least one quadrant were enrolled for the study. The investigation was evaluated and approved by the institution's ethical committee (MSRDC/EC/12-14/Perio/PG/002), and all participants provided signed informed consent.

### 2.1. Inclusion Criteria

1. 20–50 years.
2. Presence of minimum three hypersensitive teeth in at least one quadrant.
3. Hypersensitivity of teeth to tactile, cold, and air stimulation on facial aspect, who initially responded to the stimulus with a score of ≥5 in the numeric rating scale (NRS) [12, 13].

### 2.2. Exclusion Criteria

1. Previous history of professional desensitization therapy in the past 3 months.
2. Allergies and atypical reactions to milk proteins and/or hydroxybenzoates.
3. Eating disorders.
4. Excessive dietary or environmental exposure to acids.
5. History of periodontal surgery within the last 3 months.
6. Teeth with cervical restorations interfering with the evaluation.
7. Teeth covered with crowns or bridges.
8. Medically compromised patients currently using medications such as analgesics, anticonvulsant, antihistamines.
9. Breastfeeding and pregnancy.

### 2.3. Procedure

The participants were recruited from the out-patient section of Department of Periodontology, Faculty of Dental Sciences, M S Ramaiah University of Applied Sciences, Bangalore. The 39 selected patients for the study were randomized into three groups (Table 1) using a random number table. Thirteen patients allotted to Group A received 660 nm diode laser treatment, 13 patients in Group B received 980 nm diode laser treatment, and 13 patients in Group C received ACP–CPP agent (Global Chemical Public Company Limited (GC) Tooth Mousse) treatment. Two operators were involved in the study from beginning till the end. Pre- and posttreatment evaluation of DH scores on NRS, randomization and treatment group allocation (Group A, Group B, and Group C) was done by a single observer, who remained blinded to the treatment modality performed in each group. The treatment interventions were carried out by another examiner. Prior to the treatment intervention, the respondents were subjected to full mouth scaling and root planing and oral hygiene instructions were given.

### 2.4. Evaluation of DH Scores on NRS

**Figure 1:**
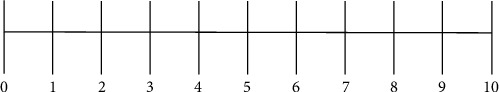
A 11-point NRS with numbers from 0 (no pain) to 10 (worst pain imaginable) used for evaluating pretreatment hypersensitivity scores and posttreatment hypersensitivity scores. NRS, numeric rating scale.

**Figure 2:**
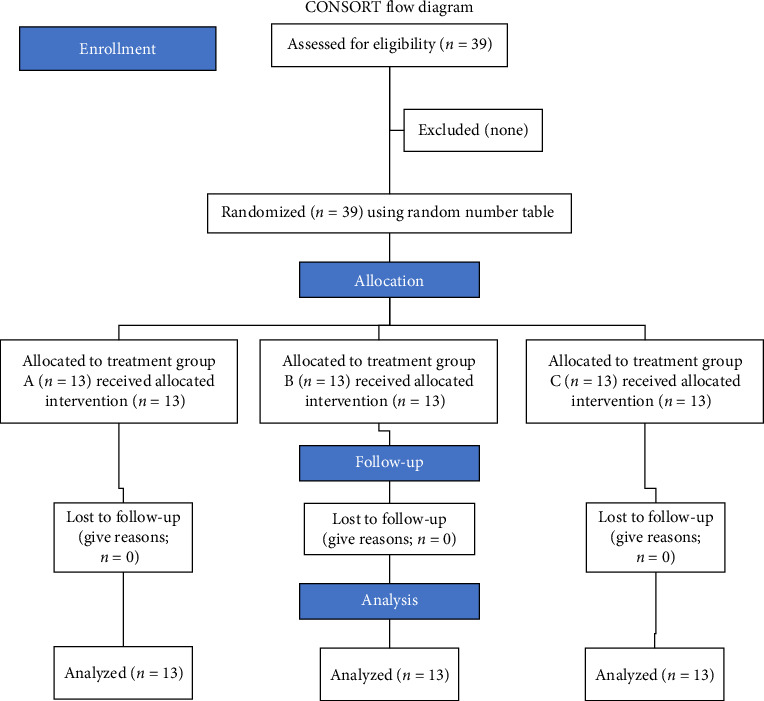
CONSORT flow diagram of study design.

All the hypersensitive teeth were stimulated with three tests: tactile stimuli—scratching horizontally along the Cementoenamel junction (CEJ) with a dental explorer, thermal stimuli—using drops of melted ice, and air stimuli—air blast from a three-way syringe at a distance of 1 cm and perpendicular to the tooth surface for 1 s. NRS (Figure 1) was used for evaluating pretreatment and posttreatment hypersensitivity scores following the application of these stimuli [12, 13]. The NRS is a scale that is commonly used to assess pain levels in medicine. It is represented on a horizontal line with an 11-point numeric range, labeled from 0 to 10, with 0 representing no pain and 10 representing the worst pain possible. It was administered via paper for the respondent to complete according to the intensity of pain perceived by them. Patients who initially responded to the tactile, cold, and air stimulus with a score of ≥5 in the NRS were involved in this research. The NRS values were recorded at baseline, 30 min after the conclusion of the three treatment sessions in the diode laser groups, 1 week, 1 month, 3 months, and 6 months.  Figure 2 represents the CONSORT flow diagram of the study.

### 2.5. Application of 660 nm Laser

The laser was used following the manufacturer's (Hager & Werken GmbH & Co KG, Germany) instructions. A 660 nm laser was irradiated on selected teeth, in a continuous wave and no contact mode at 40 mW power, using 320-micron diameter fiber. It was administered for 8 s at hypersensitive spots in three sessions separated by 48 h.

### 2.6. Application of 980 nm Laser

980 nm laser was irradiated on affected teeth at 2 W power, continuous wave and no contact mode, using a 320-micron diameter fiber. The procedure was performed in three sessions at 1-week interval for 3 weeks, and laser was administered at each site for 1 min.

### 2.7. Application of ACP–CPP Agent (GC Tooth Mousse)

Using an applicator tip, a pea-sized amount of ACP–CPP agent was applied to the tooth surface and left undisturbed for 3 min. The patient was then directed to distribute the leftover drug throughout the mouth with the tongue, avoid expectoration, and postpone swallowing for an additional 1–2 min. The patients were also informed that after treatment, “do not eat or drink” for 30 min.

### 2.8. Method of Statistical Analysis

Data were set into Microsoft Excel, then evaluated with the Statistical Package for the Social Sciences (SPSS; Version 23.0) software suite. Tables show the outcomes, which were averaged in the case of continuous data and number and percentage in the case of binary data. Shapiro–Wilk test was used to determine the normalcy of the information being analyzed. The Chi-square (*χ*^2^) test of significance was used for contrasting ratios. The Kruskal–Wallis and Mann–Whitney *U* tests were employed to compare groups. The “*p*” value of 0.05 was regarded as demonstrating statistical significance in all of the preceding tests.

## 3. Results

**Figure 3:**
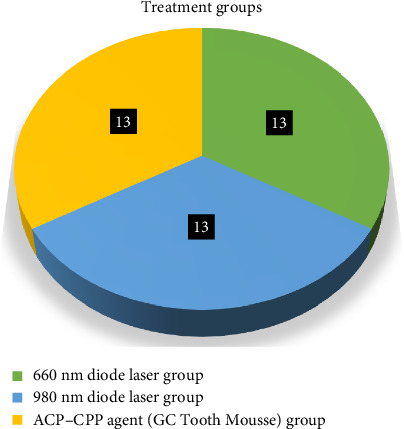
Patients allocated to each group. ACP, amorphous calcium phosphate; CPP, casein phosphopeptide; GC, Global Chemical Public Company Limited.

Total 39 respondents contributed in this research at the end of the study period (Figure 3). Thirteen patients each were randomly allocated to the three study groups.  Table 2 depicts the contrast of mean NRS scores within the treatment groups from baseline to 6 months.

**Figure 4:**
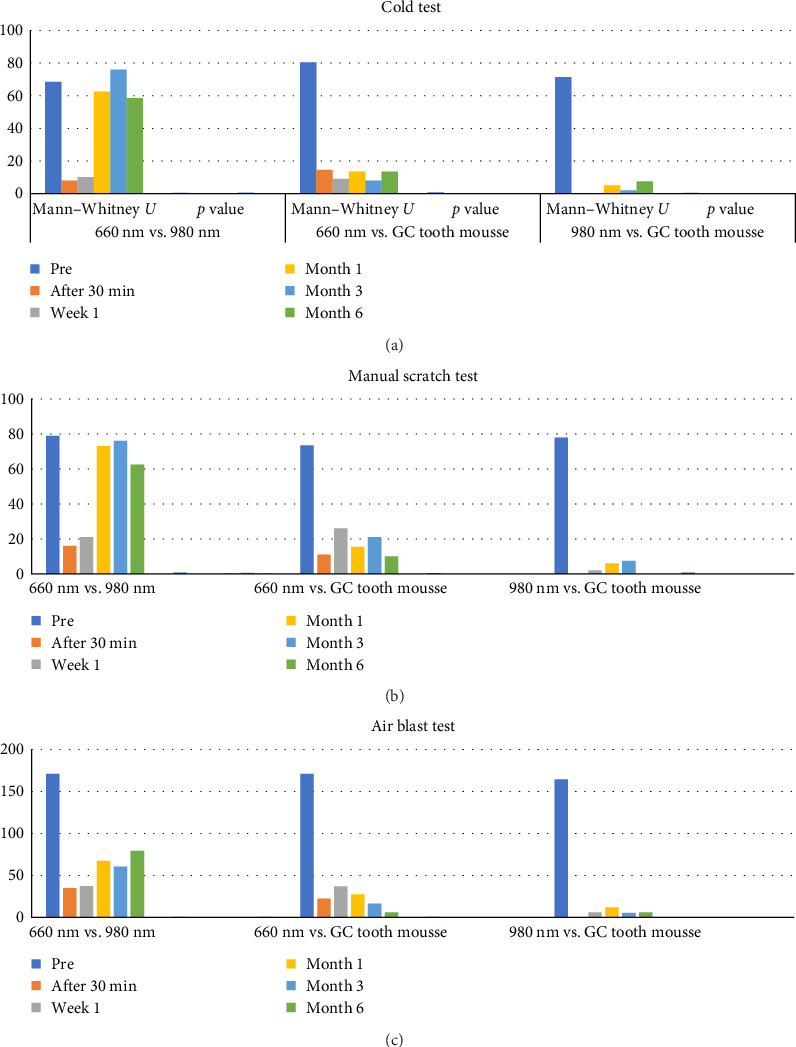
Comparison of mean NRS scores between the treatment groups at different time intervals for (a) cold test, (b) manual scratch test, and (c) air blast test. GC, Global Chemical Public Company Limited; NRS, numeric rating scale.

Comparison of mean NRS scores within the three treatment groups for cold test, manual scratch test and air blast test (Table 2) showed a statistically significant reduction in the NRS scores (*p* value <0.001) from baseline to 6 months. When the mean NRS scores between the treatment groups at different time intervals for cold test, manual scratch test, and air blast test (Figure 4) were compared, there was a statistically significant difference (*p* value <0.001) between 660 nm diode laser group and 980 nm diode laser group after 30 min, and at 1 week with the 980 nm diode laser group showing greater reduction in the mean NRS scores. However, there was no statistically significant difference between these two groups at 1, 3, and 6 months. When 660 nm diode laser group and 980 nm diode laser were compared to ACP–CPP agent group, there was a statistically significant difference (*p* value <0.001) with both the laser groups showing a greater reduction in the mean NRS scores compared to ACP–CPP agent at the various study time intervals.

## 4. Discussion

Clinically, the response of hypersensitive teeth to various stimuli varies. As a result, the ad hoc advisory committee on DH [14] recommends that an efficient hypersensitivity research should include more than one stimulus. Keeping this in mind, the present research was designed including three separate stimuli: air, cold, and tactile stimuli. According to the Council of Dental Therapeutics—American Dental Association [15], an appropriate experimental stimulus should be restricted to the area of hypersensitivity. For accurate pain assessment, along with measuring pain subjectively, the threshold of response should also be established and quantified. In accordance with these criteria, in this study three different stimuli were used and the tests were repeated thrice and the average value was calculated and noted as NRS scores.

There presently exists no perfect approach for generating and measuring cervical dentin sensitivity [16]. Majority of individuals can easily follow NRS as it does not require sharp vision or skills to perform the scoring [12]. In a systematic review comparing verbal rating scale (VRS), visual analogue scale (VAS), and NRS, it was found that NRS reported better patient acceptance than the other scales. NRS-11 was the most used version of the NRS in most clinical settings [13] and hence, an assessment using NRS was used in the current research.

In current research, in 660 nm diode laser group there was a statistically significant decrease in the sensitivity scores from reference point to 24 weeks. Low level laser therapy such as using 660 nm the diode laser operates at cellular level, enhancing cellular respiration and energy generation, therefore, promoting the creation of tertiary dentin and, as a result, closing the tubules of the dentin [17, 18]. These findings were similar to a study by de Fátima Zanirato Lizarelli et al. [19], where low intensity laser therapy (LILT) at irradiation parameters of 660 nm and 40 mW was a better therapeutic method in reducing DH compared to light emitting diode and the analgesic effect of LILT is associated with deregulation of C-fiber afferents which subsequently is a photophysical shift caused by the association between physiological light and cell. In another study by Aranha, Pimenta, and Marchi [20], five treatment modalities were compared for treating DH, that is, Gluma Desensitizer, Seal&Protect, Oxa gel, Fluoride, and LILT (660 nm/3.8 J/cm ^2^/15 mW), subsequently post contemporary therapy, after 1, 4, 12, and 24 weeks. It was found that LILT presented a gradual reduction of hypersensitivity throughout the continuation of 24 weeks as observed in present research.

Another treatment group in this study was 980 nm diode laser group. The effect of 980 nm diode laser as a desensitizing agent showed a statistically significant difference in sensitivity scores from baseline to 6 months which was in accordance with a study by Miron et al. [21], where the 980 nm galium aluminum arsenide (GaAlAs) high-level diode laser was found to be effective in reducing DH as the high-power lasers, provokes a melting behavior with dentin inorganic element crystallisation and coagulation of fluids contained in dentinal tubules. In an in vitro scanning electron microscopic (SEM) study [22], it was found that following irradiation with a 980 nm diode laser, dentinal tubules can be completely occluded at 2 W power settings, and upon irradiation, no substantial alterations in pulp tissue or dental cells were found. A study assessing the effect of low-level diode laser on the topography of dentin and symptomatic noncarious cervical lesions before composite restorations revealed that 660 nm diode lasers showed the maximum decrease in sensitivity, moreover SEM observed 660 and 970 nm reduced the width of the dentinal tubules resulting in lowered sensitivity scores [23].

The third treatment group in this study using ACP–CPP agent (GC Tooth Mousse) resulted in a statistically significant reduction in sensitivity scores as it had a quick and long-lasting desensitizing impact and proved beneficial in lowering cervical DH, which could be because the CPP–ACP duo localizes in plaque as micro-collections and induces enamel revitalization at a significantly quicker pace. In a prior study, GC Tooth Mousse, when compared to another desensitizing agent, has shown better effectiveness in relieving DH [9]. Studies done by Bhandary and Hegde [10] and Torwane et al. [11] have also reported GC Tooth mousse to be effective in reducing hypersensitivity from baseline to 2 weeks and baseline to 21 days correspondingly as observed in the present research.

An interesting SEM investigation evaluated the extent of occlusion of dentin tubules utilizing GC tooth mousse, sodium fluoride varnish, and diode laser. Similar to our results, it was found that when compared to GC tooth mousse and sodium fluoride varnish, diode laser was beneficial for the regular clinical treatment of DH [24]. Another research compared the effects of low-power diode lasers (660 and 810 nm) on DH and found that 660 nm and 810 nm diode lasers at power of 30 and 100 mW, irradiated for 2 min was beneficial in reducing DH pain [25], in accordance with our study. A recent randomized clinical study by D'Amario et al. [26] compared the effects of ozone and laser on DH reported a decrease of hypersensitivity in both the groups immediately after treatment and at 3- and 6-months following therapy, similar to the present study.

Another SEM study by Alzarooni et al. [27] which aimed to assess the impact of neodymium-doped yttrium aluminum garnet (Nd:YAG) laser, glutaraldehyde-based desensitizer (GD), or their combination on occluding dentinal tubules. They concluded that Nd:YAG laser alone and in combination with GD has superior dentinal tubule occlusion in vitro. Its clinical use in the treatment of DH may overcome the drawback of conventional treatment approaches for DH needing repeated applications to achieve continuous relief from pain since acidic diet and toothbrushing result in the continuing elimination of precipitates and surface coatings. In another randomized controlled trial by Hihara et al.[28], the study aimed to evaluate the efficacy and safety of DH treatment using a newly developed device based on a powder jet deposition. The authors concluded that, powder jet deposition therapeutic effect was most likely attributable to the deposition of a hydroxyapatite layer on the tooth surface, which would alleviate hypersensitivity for at least 12 weeks without causing severe adverse events.

In an in vitro study conducted by Jeon et al. [29], the authors hypothesized that intratubular crystals formed from the experimental materials consisted of dicalcium silicate (DCS) and tricalcium silicate (TCS) were resistant to acid. These crystals significantly reduced dentin permeability. The effect of the DCS/TCS mixture and TCS on reducing discomfort due to DH has resistance potential for acid challenge. In a double blind randomized controlled trial study by Li et al. [30] in Chinese adults, the objective was to assess the efficacy and safety of a toothpaste containing 7.5% Huaxi bioactive glass–ceramic (HX-BGC) in combating DH. HX-BGC is a novel bioactive glass–ceramic material which consists of sodium calcium phosphate (NaCaPO_4_) and hydroxyl apatite to reduce dentin tubule permeability. The authors concluded that toothpaste containing 7.5% HX-BGC demonstrated more significant effects in combating DH. Our study had a contradictory result when compared to the abovementioned study, as in our study diode lasers demonstrated a greater reduction in the mean NRS scores compared to ACP–CPP agent at the various study time intervals.

In a randomized clinical trial conducted by Naghsh et al. [31], which aimed to compare the effectiveness of Gluma and high-power 980 nm diode laser, alone or in combination, in the treatment of cervical DH. The authors concluded that 980 nm diode laser alone was more effective than the other two intervention methods for 1 month. The result of this study was in accordance with our study, where 980 nm diode laser showed better potential in the treatment of DH.

This is the first original, clinical study comparing the efficacy of two different wavelengths of diode laser and ACP–CPP agent as in-office agents for the management of DH for a 24-weeks follow-up period. However, the limitations of the study could be the lower sample size as it involved only 39 subjects and the follow up period was only for 6 months. A longer follow-up period to check the effectiveness of the in-office agents over time is suggested. Also, the clinical application of diode lasers in combination with ACP–CPP agent can be tested in future researches. In this study, there was a significant difference between 660 and 980 nm diode laser at 30 min and 1 week with a greater reduction in the 980 nm group, but there was no statistically significant difference between the two groups at 1, 3, and 6 months to the cold, air, and tactile test. The greater reduction in DH observed at 30 min and 1week in the 980 nm group could be attributed to the increased melting impact with crystallization of dentin mineral content and coagulation of fluids within the tubules of dentin.

## 5. Conclusion

660 nm diode laser, 980 nm diode laser, and ACP–CPP agent showed definite potential as effective desensitizers when used as an in-office procedure. The 660 and 980 nm diode laser showed better potential than ACP–CPP agent in reducing DH throughout the study period. 980 nm diode laser showed greater reduction in DH at 30 min and 1 week compared to 660 nm diode laser. However, long-term evaluations with more participants are necessary to determine the effectiveness of these treatment approaches.

## Figures and Tables

**Table 1:** Treatment groups.

Group	Treatment method
A	660 nm diode laser
B	980 nm diode laser
C	ACP–CPP agent (GC Tooth Mousse)

Abbreviations: ACP, amorphous calcium phosphate; CPP, casein phosphopeptide; GC, Global Chemical Public Company Limited.

**Table 2:** Comparison of NRS scores within the treatment groups from baseline to 6 months.

Visit	Group	Median	Min.	Max.	*χ* ^2^	*p* value
Cold test						

Pre	A	8.00	7	9	1.027	0.598
	B	9.00	8	9		
	C	8.00	8	9		

After 30 min	A	5.00	4	6	31.175	<0.001
	B	3.00	3	4		
	C	6.00	5	7		

Week 1	A	6.00	5	6	31.865	<0.001
	B	4.00	4	5		
	C	6.00	5	7		

Month 1	A	6.00	5	7	23.236	<0.001
	B	5.00	5	6		
	C	7.00	6	8		

Month 3	A	5.00	5	7	25.592	<0.001
	B	5.00	5	6		
	C	7.00	6	8		

Month 6	A	7.00	6	7	23.443	<0.001
	B	6.00	6	7		
	C	8.00	7	8		

Manual scratch test						

Pre	A	8.00	7	9	0.495	0.781
	B	8.00	7	9		
	C	8.00	7	9		

After 30 min	A	4.00	3	6	30.171	<0.001
	B	3.00	2	4		
	C	6.00	5	7		

Week 1	A	5.00	4	6	26.341	<0.001
	B	4.00	3	5		
	C	6.00	5	7		

Month 1	A	5.00	4	7	20.950	<0.001
	B	5.00	4	6		
	C	7.00	6	8		

Month 3	A	6.00	5	7	23.075	<0.001
	B	5.00	4	6		
	C	7.00	6	9		

Month 6	A	6.00	5	7	26.326	<0.001
	B	6.00	5	6		
	C	8.00	7	9		

Air blast test						

Pre	A	8.00	7	9	0.365	0.833
	B	8.00	7	9		
	C	8.00	7	8		

After 30 min	A	4.00	3	5	25.252	<0.001
	B	3.00	2	4		
	C	5.00	5	6		

Week 1	A	5.00	3	6	20.240	<0.001
	B	4.00	3	5		
	C	6.00	5	7		

Month 1	A	6.00	4	7	17.807	<0.001
	B	5.00	5	6		
	C	7.00	6	7		

Month 3	A	6.00	5	7	23.134	<0.001
	B	6.00	4	7		
	C	7.00	7	8		

Month 6	A	6.00	5	7	24.565	<0.001
	B	6.00	5	7		
	C	8.00	7	9		

Abbreviations: *χ*^2^, chi-square; NRS, numeric rating scale.

## Data Availability

The corresponding author will provide access to the data of this study upon request.
